# The importance of being CAFs (in cancer resistance to targeted therapies)

**DOI:** 10.1186/s13046-022-02524-w

**Published:** 2022-11-03

**Authors:** Sabrina Rizzolio, Silvia Giordano, Simona Corso

**Affiliations:** 1 Candiolo Cancer Institute, FPO-IRCCS, Candiolo, Italy; 2 Department of Oncology, University of Torino, Torino, Italy

**Keywords:** CAF, targeted therapy, resistance, tumor microenvironment

## Abstract

In the last two decades, clinical oncology has been revolutionized by the advent of targeted drugs. However, the efficacy of these therapies is significantly limited by primary and acquired resistance, that relies not only on cell-autonomous mechanisms but also on tumor microenvironment cues. Cancer-associated fibroblasts (CAFs) are extremely plastic cells of the tumor microenvironment. They not only produce extracellular matrix components that build up the structure of tumor stroma, but they also release growth factors, chemokines, exosomes, and metabolites that affect all tumor properties, including response to drug treatment. The contribution of CAFs to tumor progression has been deeply investigated and reviewed in several works. However, their role in resistance to anticancer therapies, and in particular to molecular therapies, has been largely overlooked. This review specifically dissects the role of CAFs in driving resistance to targeted therapies and discusses novel CAF targeted therapeutic strategies to improve patient survival.

## Background: being CAFs

Fibroblasts and their activated counterpart resident inside the tumor mass, named cancer-associated fibroblasts (CAFs), are very enigmatic cells. Fibroblasts are extremely versatile: they are usually quiescent, but upon tissue damage and wound healing response they can be reversibly activated (‘myofibrobasts’) (reviewed in [1]). In cancers (the ‘wounds that never heal’ [2]), this activated status becomes exacerbated and irreversible, as consequence of epigenetic changes [3, 4]. Compared to normal fibroblasts, CAFs show increased proliferation and motility, as well as elevated secretion of growth factors, chemokines, and extracellular matrix (ECM)-degrading enzymes such as metalloproteases. Thus, in many experimental contexts, CAFs appear as positive regulators of tumorigenesis and metastasis [5, 6]. CAFs also contribute to the generation and maintenance of the cancer stem cell ‘niche’ through the active remodeling of ECM and secretion of morphogens [7, 8]. CAFs regulate ferroptosis in surrounding tumor cells [9] and they also develop metabolic symbiosis with cancer cells, mutually and dynamically reprogramming their basal metabolism- comprising lipid metabolism [10, 11] - in surrounding tumor cells to generate a pro-tumorigenic ecosystem [12]. CAFs do not only interact with tumor cells, but they are functionally connected also with other cells in the tumor microenvironment, including vascular endothelial cells and immune cells. Indeed, CAFs secrete factors that modulate vascular network formation/ remodeling [13–15] and they deeply influence the functions of several immune cell types, including macrophages, neutrophils and T cells [16]. In this context, several authors reported that CAFs can promote an immunosuppressive environment, both directly, through the secretion of several chemokines or other negative immune-regulators [17, 18], and indirectly, by regulating the stiffness of the ECM, which decreases immune cell infiltration or immune cell extravasation [19].

Interestingly, it is emerging that CAFs (as well as myofibroblasts) are highly heterogeneous cells with distinct gene expression patterns and different, sometimes opposite, biological functions inside the tumor microenvironment (TME) [20–23]. Even in the same tumor, different CAF subpopulations can be present. In pancreatic ductal adenocarcinoma (PDAC), Öhlund et al. have identified two spatially separated, reversible, and mutually exclusive subtypes of CAFs: myCAFs (myofibroblastic CAFs), closely bound to cancer cells and characterized by high αSMA expression, and iCAFs (inflammatory CAFs), located more distantly from neoplastic cells, which are characterized by significantly lower αSMA levels and elevate expression of cytokines with known roles in cancer progression, such as IL-6 and IL-1 [20]. Moreover, a third CAF subtype has been identified, named apCAFs (antigen-presenting CAFs), expressing MHC II genes [24], deriving from mesothelial cells [25] and promoting or suppressing immune response, depending on the tumor context [25, 26]. Accordingly, recent studies have shown that, in certain contexts, CAFs may act as negative regulators of tumor progression, restraining, rather than supporting, pancreatic ductal adenocarcinoma growth [27, 28]. This has been clearly shown in two different experimental models: (i) transgenic mice developing spontaneous PDAC crossed with alpha smooth muscle actin (αSMA)-tk transgenic mice to selectively target αSMA+ myofibroblasts upon ganciclovir administration [27] or ii) conditional deletion of Sonic Hedgehog, the key factor driving formation of a fibroblast-rich desmoplastic stroma in PDAC [28]. The derived pancreatic tumors, bearing a reduced stromal content, were more undifferentiated, vascularized, and aggressive. The increased aggressiveness was either due to suppressed immune surveillance [27] or to altered angiogenesis [28], suggesting that CAFs can negatively control tumor growth by negatively controlling the Treg repertoire, and restraining tumor angiogenesis. Recently, through single-cell mass cytometry, Hutton et al. [29] uncovered two fibroblast lineages with opposite effects on PDAC progression. The two cell subsets, identified both in normal and in cancer tissues, were stably demarked by the expression CD105, a co-receptor for the TGFb family ligands: CD105 positive fibroblasts gave rise to tumor permissive CAFs, while CD105 negative fibroblasts differentiated into CAFs with tumor suppressive properties, by supporting anti-tumor immunity. Similarly, two distinct CAF populations with opposing roles in the progression and immune landscape were identified in PDAC, as, in this context, depletion of fibroblast activation protein (FAP)+ CAFs increased survival, while depletion of αSMA+ CAFs decreased survival [30]. Also the TGFβ-driven expression of the leucine-rich-repeat-containing protein 15 (LRRC15) in CAFs, characterizes a pro-tumorigenic CAF subpopulation, as the depletion of LRRC15+ CAFs in PDAC models slowed tumor growth and restored CD8+ T cell functions, increasing response to immunotherapy [31]. Why CAFs are so heterogeneous is not clear. One possible explanation is the source of origin: indeed, studies performed in genetically modified animals suggest that CAFs can derive not only from resident fibroblasts, but also from bone marrow cells [32], adipocytes [33] or epithelial cells undergone mesenchymal transition [34].

Finally, robust evidence has indicated that CAFs play a major role in drug resistance. In this review we will focus on CAF role in resistance to targeted agents, while stroma-mediated resistance to chemo-, radio-, or immunotherapies has been nicely reviewed elsewhere [16, 35].

### Limitation of preclinical models to understand CAF biology

A general and important premise concerning studies of CAF-mediated drug resistance is the limitation of reliable preclinical models. *In vitro* models frequently used to evaluate the CAF activity include direct co-culture of tumor cells and CAFs, indirect co-culture systems (i.e., co-culture separated by a filter), or treatment with conditioned media. Notably, murine CAFs can be easily obtained and propagated in culture from human xenografts. Diphtheria toxin, that selectively kills human but not mouse cells, can be used to isolate the mouse CAF population [36, 37]. It is more difficult to obtain human CAFs stably growing *in vitro*, especially from very small samples. Hu et al. recently succeeded in establishing a large collection of CAFs derived from non-small cell lung cancer (NSCLC) biopsies by immortalizing early derived CAF cultures with human telomerase reverse transcriptase, thereby preventing senescence [38]. The authors demonstrate that these immortalized CAFs maintain the expression profile of their parental counterparts and can be efficiently used in preclinical studies [38]. The use of established CAF cultures allows for molecular perturbations, such as CRISPR gene editing and reliable repetition of experiments. However, while working with CAFs *in vitro*, particular attention should be paid to the culture conditions, as both serum and stiff substrates are able to modulate fibroblast activation, possibly changing the original CAF features. 3D culture models, that is organoids containing fibroblasts and immune components (‘organoids 2.0’) have been recently developed and recapitulate TME diversity, offering great promise for *in vitro* modelling of personalized immunotherapy [39, 40]. However, it should be considered that in these 3D models, the basement membrane preparations in which they are embedded often contain a standard growth factor mix, in addition to matrix components, that may alter CAF biology.

The models that best recapitulate the crosstalk between CAFs and tumor cells are those *in vivo*, namely genetically engineered mouse models (GEMM), tumor xenografts and patient-derived xenografts (PDXs). In these last two models, human CAF functions can be explored *in vivo* through co-injection of CAFs and tumor cells. However, in this case tumors contain human CAFs mixed with mouse-derived fibroblasts, that usually outgrow the injected CAFs, making it difficult to test long-term biological properties such as responses to therapy.

All these issues should be carefully evaluated when considering the real clinical relevance of studies on CAF-mediated resistance.

### How do CAFs mediate resistance to anti-cancer therapy?

In addition to the well-studied cell-autonomous resistance escape routes (e.g., oncogene mutations, activation of bypass signaling pathways, epigenetic modifications), in the last decade also ‘non-cell-autonomous’ mechanisms of drug resistance have emerged, with CAFs often being crucial mediators of resistance to targeted agents. How do they mediate resistance to molecular therapies? It is clear that they can do it in several ways, through the ECM components they produce, the soluble factors and extracellular vesicles they release, and even their metabolism. Besides the direct effects that CAFs exert on tumor cells, we have to consider that CAFs can also indirectly modulate drug response through a complex network of interactions with other cells of the TME, for example through modulation of tumor angiogenesis and immune response. Concerning the effect on vessels, CAFs have been reported to induce chemoresistance by promoting microvessel leakiness in ovarian cancer [41], opening the possibility that this mechanism might alter the delivery of molecular compounds as well. Concerning the effect on the immune compartment, CAFs not only influence response to immunotherapy [18, 42] but might indirectly influence the response to targeted therapies, as many targeted compounds have additional effects on the immune system that contribute to their therapeutic efficacy [43].

#### The role of the extracellular matrix

**Fig. 1:**
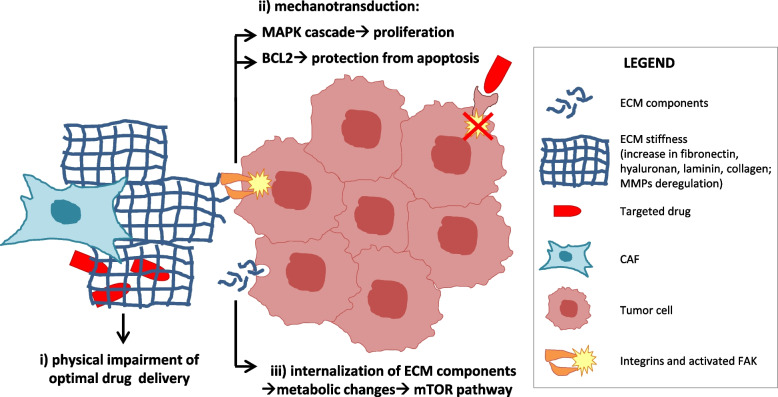
Matrix-mediated resistance to targeted therapies. Major mechanisms of resistance to molecular therapies mediated by CAF matrix are depicted. ECM: extracellular matrix; MMPs: matrix metalloproteinases; FAK: focal adhesion kinase

Stiffness is a biophysical property of the ECM that affects several cellular functions, including proliferation, invasion, differentiation, and also therapeutic responses. The increased production of ECM components characterizes the transition from normal to activated fibroblasts, thus representing a typical trait of CAFs. Indeed, the biophysical properties of the tumor matrix progressively change during tumor progression and can be further modulated by cancer therapies. In particular, both chemotherapy and radiotherapy can drive strong matrix remodeling, pushing local CAFs to revise their secretion of fibers, glycoproteins, fibronectin or enzymes responsible for ECM post-translational modifications, eventually leading to tumor desmoplasia that blunts therapeutic efficacy [44]. Changes in the biochemical and biomechanical matrix properties can also contribute to resistance to targeted agents (Fig. 1). For example, intra-vital imaging of BRAF-mutant melanoma cells containing an ERK/MAPK biosensor revealed how the extracellular matrix affected the response to the BRAF inhibitor PLX4720 [45]. Even though at first melanoma cells responded to PLX4720, rapid MAPK signaling reactivation was observed in areas of high stromal density. This was linked to fibroblast “paradoxical” activation by PLX4720 and the subsequent promotion of matrix production and remodeling, resulting in elevated integrin β1/FAK/Src signaling in melanoma cells. Indeed, fibronectin-rich matrices were able to elicit PLX4720 tolerance and, conversely, addition of FAK inhibitors to PLX4720 prevented the onset of resistance to the BRAF inhibitor. Thus, activated fibroblasts and the rigidity of the matrix provide a sanctuary for melanoma cells to survive BRAF targeting [45].

Increased matrix rigidity induced by YAP/TAZ activation also led to resistance to the HER2 tyrosine-kinase inhibitor (TKI) lapatinib in *HER2*-amplified breast cancer cells when cultured on substrates engineered to mimic different levels of matrix rigidity [46]. Using a three-dimensional co-culture model, Marusyk et al. demonstrated that the spatial proximity of breast ductal carcinoma cells to CAFs contributes to lapatinib resistance, which is partly mediated by hyaluronan [47]. Indeed, when tumor cells were embedded in Matrigel in the presence of CAFs and treated with lapatinib, drug accumulation was reduced compared to tumor cells cultured without CAFs; these results were validated in *in vivo* models as well. Consistent with the reduced intracellular accumulation of the drug, the effect of lapatinib on HER2, EGFR, and AKT phosphorylation was less pronounced, and apoptosis was attenuated, as shown by reduced cleaved caspase-3 levels. Notably, protection from lapatinib requires close physical proximity between fibroblasts and carcinoma cells, and hyaluronidase treatment completely abolished the protective effect of stromal fibroblasts both *in vitro* and *in vivo,* indicating that, in this context, hyaluronan is essential for sustaining resistance to lapatinib [47].

In addition to hyaluronan, other ECM components, such as laminin, may affect the sensitivity of breast ductal carcinoma to lapatinib. Indeed, tumor cells in niches with laminin-enriched ECM express more anti-apoptotic Bcl-2 family proteins and exhibit resistance to lapatinib [48]. Similarly, elevated deposition of laminin-5 in breast tumors conferred resistance to anti-HER2 compounds (lapatinib and the HER2 monoclonal antibody trastuzumab), through the activation of an integrin-CD151-FAK mediated pathway [49].

Collagen type I, one of the major tumor ECM components, was also involved in resistance to molecular therapies. In triple-negative breast cancer, the efficacy of the multi-kinase inhibitor sorafenib, was reduced in collagen-rich microenvironments, due to JNK signaling activation [50]. In another model, collagen was also responsible for resistance to EGFR inhibitors, even if through a different mechanism [51]. Indeed, in this context, collagen I was internalized by tumor cells through RAC1-mediated micropinocytosis, and catabolized. The derived aminoacids, mainly prolin and hydroxyprolin, affected cellular metabolism and induced mTOR activation and drug resistance. Consistently, both macropinocytosis and RAC1 inhibitors prevented resistance to the EGFR TKI gefitinib [52]. Since other major ECM components, such as laminin and fibronectin, are usually uptaken by cancer cells [53, 54] this could represent a more general mechanism of drug resistance.

Integrin β1-overexpressing cells showed increased adhesion to collagen or fibronectin [55], and the reciprocal activation of integrin β1 and EGFR was reported to mediate resistance to EGFR TKIs in several contexts [56, 57]. Even if, in the majority of the above-cited works, the Authors did not formally demonstrate the involvement of CAFs in the production of the ECM components driving resistance, the role of the CAFs is at least highly probable, since they are the main source of these components in the TME. Finally, given the role of ECM composition in drug response, it is expected that matrix metalloproteinases (MMPs) could play a role in resistance as well, as they are the main enzymes involved in ECM remodeling [58]. However, while many authors reported a role of MMPs in resistance to chemotherapy, few data are currently available for targeted therapy. In particular, in head and neck squamous cancers, response to the EGFR monoclonal antibody cetuximab was influenced by CAF-produced matrix metalloproteinase1 (MMP1) [59]. When co-cultured, both tumor cells and fibroblasts upregulated MMP1, while MMP1 inhibitors/silencing restored the response to cetuximab, further supporting the importance of proper matrix stiffness for the optimal response to molecular therapies.

Altogether, it appears that the composition of ECM can alter the response to targeted therapies in several manners (summarized in Fig. 1): i) through the physical impairment of optimal drug delivery due to increased matrix rigidity; ii) by integrin-mediated activation of pro-mitogenic and/or anti-apoptotic pathways (‘mechanotransduction’) or iii) through metabolic changes in tumor cells due to internalization of ECM components. These mechanisms have been reported in separate models, but it is conceivable that they could act also simultaneously.

#### The role of soluble factors

**Fig. 2:**
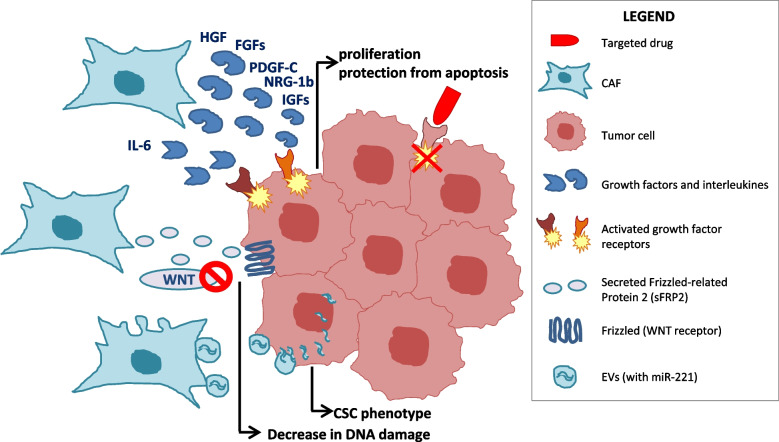
Resistance to targeted therapies: the role of soluble factors. Main mechanisms of resistance to molecular therapies mediated by CAF-produced soluble factors and exosomal vescicles are represented. HGF: Hepatocyte Growth Factor; FGF: Fibroblast Growth Factor; IGFs: Insulin-like Growth Factors; PDGF-C: Platelet-Derived Growth Factor C; NRG1b: Neuregulin-1b; IL-6: interleukin 6; sFRP2: secreted frizzled related protein 2; EV: exosomal vesicles; CSC: cancer stem cell

CAFs release an abundant secretome, mainly consisting of growth factors and cytokines that either directly or indirectly regulate tumor growth, survival, and drug response (Fig. 2 and Table 1). Recently, through *in vitro* and *in vivo* experiments, Hu et al. identified three functionally distinct subtypes of lung CAFs that are differentially able to affect the therapeutic efficacy of EGFR or ALK inhibitors in NSCLCs [38]. These three subtypes are mainly defined by the expression levels of two growth factors: hepatocyte growth factor (HGF), the ligand of the MET receptor, and fibroblast growth factor 7 (FGF7), whose major receptor is FGFR2. Subtype I CAFs secrete high levels of HGF (with or without FGF7 overexpression) and confer resistance to EGFR and ALK inhibitors; subtype II CAFs release low levels of HGF but high levels of FGF7 and confer modest resistance to EGFR and ALK inhibitors; subtype III CAFs, that produce low levels of these two growth factors, lack any protective activity against EGFR/ALK inhibitors and are associated with immune cell recruitment, suggesting a possible tumor response to immunotherapy. Notably, FGF family members and HGF were identified as the most abundant factors in CAF supernatants, and were able to confer resistance to lapatinib treatment to advanced esophageal squamous cell carcinoma (ESCC) cells [60], extending their role beyond lung cancer. HGF is one of the growth factors most implicated in resistance onset *via* stromal regulation. In two pivotal studies published 10 years ago, HGF was shown to mediate resistance to different molecular therapies in tumor cells of different origins [61, 62]. In particular, in BRAF-mutated melanomas, CAF-produced HGF was able to activate the MAPK and AKT pathways in tumor cells, thus compensating for BRAF switch-off and sustaining resistance. Immunohistochemical (IHC) analysis of BRAF V600E melanoma patient-derived biopsies highlighted that patients with abundant stromal HGF showed a poorer response to BRAF inhibitors than those lacking stromal HGF [61]. In agreement with this finding, an increase in plasma HGF was associated with worse outcomes in a cohort of patients with BRAF-mutant metastatic melanoma [62]. However, in subsequent studies, IHC detection of stromal or tumor HGF in pre-therapy melanoma specimens failed to predict patient response to BRAF inhibitors [63]; therefore, the power of HGF as a negative predictor of response to BRAF-targeted therapies needs to be further investigated.

**Table 1:** CAF-secreted soluble factors involved in resistance to targeted therapies

CAF-secreted soluble factors	Mechanism of resistance to targeted therapies	Clinical application of inhibitors: representative agents in phase2/3 clinical trials
Hepatocyte Growth Factor (HGF)	Activation of MET anti-apoptotic and pro-mitogenic downstream pathways in tumor cells Induction of stabilization/upregulation of multiple EGFR binding partners such as Axl, EphA2, CDCP1, JAK1 and integrin Beta-4	*MET (HGFR) TKIs:* Foretinib (GSK1363089) Crizotinib (PF-02341066) Cabozantinib (BMS-907351) Capmatinib (INC280) Tepotinib (EMD 1214063) *HGF targeting mAbs:* Rilotumumab (AMG 102) Ficlatuzumab (AV-299) L2G7 (TAK-701)
Fibroblast Growth Factors (FGFs)	Activation of FGF Receptors (mainly FGFR2) and their anti-apoptotic and pro-mitogenic downstream pathways in tumor cells	*Pan-FGFR TKIs:* Erdafitinib (JNJ-42756493) Derazantinib (ARQ087) Rogoratinib (BAY1163877) Dovitinib (TKI258) AZD4547 Futibatinib (TAS-120) Zoligratinib (Debio-1347) Infigratinib (BGJ398)
Transforming Growth Factorβ (TGFβ)	Upregulation of lncRNAs, including the lncRNA HOTAIR, able to activate estrogen receptor function in the absence of estrogens	*TGFβ Receptor inhibitors:* Galunisertib (LY2157299) *TGFβ Receptor mAbs:* Fresolimumab (GC1008) *TGFβ antisense oligonucleotides:* Trabedersen (AP 12009)
Neuregulin-1b (NRG-1b)	Increased expression of FOXA1 and HER3 in cancer cells; HER3 activation.	No inhibitors in phase 2/3 trials
Insulin Growth Factor 2 (IGF2)	Activation of IGF1R anti-apoptotic and pro-mitogenic downstream pathways in tumor cells	*IGF-1R TKIs:* Linsitinib (OSI-906) Ceritinib (LDK378) Brigatinib (AP26113)
Platelet-Derived Growth Factor C (PDGF-C)	Activation of PDGFR and promotion of angiogenesis	*PDGFR-α inhibitors:* Imatinib (STI571) Ponatinib (AP24534) Nintedanib (BIBF 1120) Crenolanib (CP-868596) Masitinib (AB1010)
IL-6 family members	Expansion of the stem cell pool via JAK1/STAT3 signaling Activation of NF-kB and AKT pathways in cancer cells	*IL-6 targeting mAb:* Siltuximab (CNTO 328) *JAK1/2 inhibitors:* Ruxolitinib (INC424, INCB1842)
Chemokine (C-X-C motif) ligand 13 (CXCL13)	Recruitment of B lymphocytes that produce pro-survival cytokines	No inhibitors in phase 2/3 trials
Secreted Frizzled Related Protein 2 (sFRP2)	Wnt Antagonist, Loss Of The Key Redox Effector APE1 And Attenuated Response To ROS-Induced DNA Damage	No inhibitors in phase 2/3 trials

In a screening of tumor cell lines derived from breast, kidney, liver, and tongue carcinomas, HGF conferred resistance to EGFR inhibitors by inducing the stabilization/upregulation of multiple EGFR binding partners such as Axl, EphA2, CUB domain-containing protein1 (CDCP1), JAK1 and integrin Beta-4 [64]. Importantly, the combined use of gefitinib and an anti-HGF antibody or antagonist successfully overcame fibroblast-induced EGFR-TKI resistance both *in vitro* and *in* vivo. Similarly, HGF secreted by fibroblasts was implicated in lung cancer resistance to irreversible EGFR inhibitors [65] and protected tumor cells from EGFR inhibitors in breast cancer cells bearing EGFR overexpression [66].

A recent study by our group revealed a HGF-mediated metabolism-based mechanism of non-cell-autonomous secondary resistance to MET and EGFR inhibitors [37]. In *in vivo* models of adaptive resistance to MET or EGFR TKIs, we found that resistant cells underwent metabolic reprogramming towards aerobic glycolysis, resulting in increased lactate production. This instructed CAFs to over-secrete HGF, that activated the MET pathway in tumor cells, thus favoring their escape from MET or EGFR targeting. Consistently, either pharmacological or genetic targeting of lactate metabolism, as well as concomitant MET-EGFR blocking, were able to overcome resistance. Accordingly, increased production of stromal HGF was detected in the stroma of lung cancer patients upon the emergence of resistance to EGFR TKIs, thus corroborating the clinical relevance of the reported findings [37].

CAF-derived HGF is also causally involved in resistance to anti-EGFR monoclonal antibodies. In colorectal ‘xenospheres’ treated with cetuximab, CAF-produced HGF significantly protected colon cancer stem-like cells from the effect of the drug, by preserving cell viability and inhibiting apoptosis; *in vivo*, the concomitant inhibition of EGFR and MET resulted in a more pronounced tumor regression compared to cetuximab monotherapy [67]. Consistently, in a public dataset of human, KRAS wt, metastatic colorectal cancer patients, HGF expression was significantly higher in cetuximab non-responders than in responders [67]. Notably, in a prospective trial evaluating genomic and transcriptomic determinants of resistance to cetuximab, Woolston et al. found no genetic driver of acquired resistance in a large fraction (9 out of 14, 64%) of metastases biopsied from relapsed patients. However, the majority of these biopsies showed a transcriptional switch towards a fibroblast- and growth factor-rich subtype, further supporting the idea that adaptive non-cell-autonomous mechanisms could play a relevant role in the onset of mAb resistance. Notably, also in this case, the growth factors upregulated in cetuximab-resistant biopsies were HGF and FGFs, as well as TGF-β1 and -β2 [68]. TGFβ is another cytokine abundantly released by CAFs that regulates several cancer-related pathways and plays an important role in tumor progression [69]. TGFβ also drives the upregulation of several long non-coding RNAs (lncRNAs), including the lncRNA HOTAIR, that is upregulated in tamoxifen-resistant breast cancer, where it activates estrogen receptor function in the absence of estrogen, leading to tamoxifen resistance [70]. In breast cancer, CAF-produced FGF5 was causally involved in resistance to HER2 targeted therapies (both TKIs and monoclonal antibodies) by activating FGFR2 and c-Src downstream pathways. In agreement with these preclinical data, combined elevated expression of FGF5 and phospho-HER2 correlated with a reduced pathologic response in patients treated with trastuzumab-based neoadjuvant therapy [71].

In addition to HGF and FGFs, other soluble factors secreted by CAFs have been implicated in tumor resistance to molecular therapies. In agreement with what was previously shown by Wilson et al. [62], in HER2+ breast cancers, Neuregulin-1b suppressed the response to anti-HER2 compounds through increased expression of the transcription factor forkhead box protein A1 (FOXA1) and HER3 [72]. A role of CAF-derived Neuregulin 1 (NRG1) in drug resistance was also reported by Zhang et al, who demonstrated that this soluble molecule conferred anti-androgen resistance in prostate cancer, again through HER3 activation, and that patients with increased tumor NRG1 activity showed a lower response to second-generation antiandrogen therapy [73].

In cholangiocarcinomas treated with EGFR inhibitors, a positive loop between CAF-produced IGF2 and IGF1R expressed by tumor cells was responsible for resistance to the EGFR TKI erlotinib; in line, a combined regimen of EGFR and IGF1R inhibitors overcame resistance in cholangiocarcinoma xenografts and reduced their stromal content [74]. Interestingly, IGF1 is also a key player in mediating crosstalk between KRAS G12D mutated pancreatic cancer cells and their surrounding stroma. Indeed, KRAS mutated tumor cells induced stromal cells to secrete IGF1 and GAS6 that in turn activated IGF1R and AXL signaling in tumor cells, leading to increased mitochondrial performance, proliferative capacity, and resistance to apoptotic stimuli [75]. Finally, CAFs mediated resistance to VEGF inhibitors in lymphoma xenografts models, by reactivating angiogenesis through platelet-derived growth factor C (PDGF-C) signaling, and PDGF-C targeting showed additive effects with anti-VEGFA antibodies [76].

CAFs are known to produce a number of cytokines and chemokines [27, 77] whose causative relationship with resistance to cancer therapies is well established. For example, Shein K. and colleagues found that CAF-released IL-6 family members mediated NSCLC acquired resistance to EGFR TKIs in a JAK1/STAT3–dependent manner [78]. In breast cancer, CAF-produced IL-6 acts in a paracrine manner on cancer cells, inducing expansion of the stem cell pool via JAK1/STAT3 signaling and evasion from targeted therapy [79] . IL-6 sustains resistance also through the NF-kB and AKT pathways. Gene set analysis in patients showed that high IL-6 and NF-kB expression levels correlated with poor overall survival [79]. CAF-produced cytokines could also indirectly mediate resistance; for example, CAF-derived CXCL13 promotes the recruitment of B lymphocytes into androgen-deprived prostate tumors; these prostate-cancer infiltrating lymphocytes produce other cytokines, such as lymphotoxin, promoting survival and proliferation of castration-resistant prostate cancer initiating cells, ultimately resulting in hormone resistance [80]. The ability of CAFs to confer drug resistance might be also related to their age. Spheroids treated with medium derived from ’young’ fibroblasts (i.e derived from <35-year-old donors) were more sensitive to BRAF inhibitors than those exposed to ‘aged’ fibroblasts (i.e from >55-year-old donors) medium. *In vivo*, tumors grown in 8-week-old mice responded to PLX4720 more robustly than those developed in 52-week-old mice. The molecular interpretation is that aged fibroblasts secrete a Wnt antagonist, sFRP2, which activates a multistep signaling cascade in melanoma cells, resulting in a decrease in β-catenin/MITF activity and in loss of the key redox effector APE1. Loss of APE1 attenuates the response of melanoma cells to ROS-induced DNA damage, rendering them more resistant to targeted therapy [81].

Finally, recent studies have shown that the CAF ‘secretome’ also includes exosomal vesicles that can convey paracrine signals to cancer cells, eventually regulating drug response (Fig. 2). CAF exosomes can incorporate miRNAs, functional DNA fragments, cytokines and growth factors, that are responsible for tumor progression and resistance to chemotherapy in several contexts (reviewed in [82, 83]). Concerning their role in resistance to molecular therapies, Sansone and colleagues demonstrated that CAFs can sustain hormonal therapy resistance in luminal breast cancer through the release of miR-221 containing exosomes; the horizontal transfer of this microRNA to cancer cells pushed them towards a cancer stem cell (CSC) phenotype, resistant to therapy. In line, CAF depletion restored sensitivity to hormonal therapy, with a concurrent reduction in CSCs [84]. In general, CAF paracrine signaling through exosomes seems to promote the expansion of subpopulations with stem cell features, resistance to therapy, and re-initiation of tumor growth [85]. We can foresee that the role of exosomes in resistance to targeted therapies will emerge more and more in the near future.

#### The role of metabolic changes

**Fig. 3:**
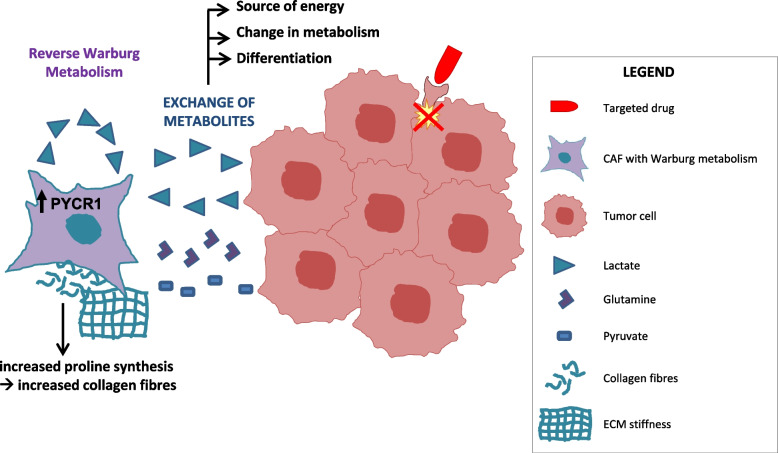
Metabolic resistance to targeted therapies. Main mechanisms of resistance to molecular therapies based on CAF/tumor cell metabolic coupling are reported

As previously mentioned, most studies on the reciprocal interaction between CAFs and tumor cells focused on the structural support provided by the CAF matrix and the pro-mitogenic/anti-apoptotic properties conferred by CAF-released growth factors. However, several studies have also highlighted the functional role of CAF/cancer cell metabolic coupling in regulating different tumor properties, including drug resistance (Fig. 3).

During tumor progression, CAFs frequently undergo a metabolic switch towards aerobic glycolysis (the so-called Reverse Warburg Effect [86]), resulting in the secretion of energy-rich metabolites that are then captured by cancer cells to fuel their anabolic metabolism [87–89].

As previously mentioned, we demonstrated that during treatment with MET or EGFR TKIs, cancer cells underwent a metabolic switch and increased lactate production, thus instructing CAFs to produce resistance-promoting growth factors [37]. In the same resistant tumors, we observed that the metabolic switch was not restricted to cancer cells but also occurred in CAFs, that showed features of enhanced glycolytic metabolism. This ‘Reverse Warburg metabolism’ allowed CAFs to indefinitely maintain HGF overexpression in culture, even in the absence of cancer cells [37].

CAF metabolism also affects the response to tamoxifen in ER+ breast cancers. When ER+ breast cancer cells were co-cultured with fibroblasts, reactive oxygen species (ROS) produced by tumor cells in response to tamoxifen treatment drove aerobic glycolysis in fibroblasts; the excess of lactate produced by CAFs induced mitochondrial biogenesis in the adjacent tumor cells, forcing them to switch towards an oxidative state; this metabolic state, with glycolytic CAFs fueling the oxidative tumor cells, sustained anabolic growth and tumor survival in the presence of tamoxifen [90]. Interestingly, Eckert et al. identified methyltransferase nicotinamide N-methyltransferase (NNMT) as a master metabolic regulator of CAFs in ovarian cancer, epigenetically controlling widespread gene expression changes in the TME during tumor progression [91]. In prostate adenocarcinoma cells, increased CAF glutamine production due to epigenetic silencing of the RAS inhibitor RASAL3 serves as a source of energy and as a mediator of neuroendocrine differentiation, ultimately leading to resistance to androgen signaling deprivation therapy (ADT). In agreement with these findings, prostate cancer patients resistant to ADT showed elevated blood glutamine levels compared with those with therapeutically responsive disease; antagonizing stromal glutamine uptake was sufficient to restore ADT sensitivity in castration-resistant xenograft models [92].

The ‘Reverse Warburg’ could be induced in CAFs by breast cancer cells through the abnormal activation of an estrogen/GPER/cAMP/PKA/CREB signaling axis; glycolytic CAFs, in turn, fed tumor cells with extra pyruvate and lactate, increasing mitochondrial activity and conferring breast cancer cells with drug resistance to several conventional clinical treatments, including endocrine therapy, HER2 targeting and chemotherapy [93].

Finally, CAF metabolism directly influences ECM composition: the production of massive amounts of collagens by activated fibroblasts requires increased proline synthesis from circulating glutamine, and this relies on increased expression of pyrroline-5-carboxylate reductase 1 (PYCR1) in CAFs, which is in turn epigenetically regulated by histone acetyl-transferase EP300 and by acetyl-CoA levels [94]. This was demonstrated in detail in breast cancer models, but PYCR1 and collagen upregulation co-occurs in many tumor types [94], suggesting that this mechanism might have a broader relevance. As collagen abundance and ECM stiffness drive therapeutic resistance, these findings might represent another way by which metabolic cues influence drug response.

### Therapeutic opportunities

Given their relevant role in mediating or accelerating the onset of drug resistance, their abundance in the tumor microenvironment, and their genetic stability, CAFs are now considered appealing targets for anticancer therapeutic strategies. However, several challenges are currently present in our attempts to modulate CAFs for therapeutic benefit, *in primis* the shortage of CAF-specific markers. Even the most widely used CAF markers, such as fibroblast activating protein (FAP) and α-Smooth Muscle Actin (αSMA) are not exclusive of CAFs; indeed, FAP is expressed also in smooth muscle and epithelial cells while αSMA is present in smooth muscle cells, pericytes and myoepithelial cells. Another big challenge is represented by the heterogeneity of CAF functions, which, as described above, can be either tumor-promoting or tumor suppressive, depending on the context [20, 25–28]. Also in relation to drug resistance, different CAF types can drive tumor sensitivity or resistance to the same therapy. Brechbuhl et al. demonstrated that in ER+ breast cancers, CD146^-^ CAFs suppressed ER expression, thus decreasing tumor cell sensitivity to estrogen and increasing resistance to tamoxifen, whereas CD146^+^ CAFs promoted ER expression, sustaining estrogen-dependent tumor proliferation and tamoxifen sensitivity [95].

In this scenario, indiscriminate targeting of the whole CAF population could be ineffective or even harmful, thus making it necessary and urgent to identify reliable markers of the two subpopulations. In this context, two recent works offered great expectations [29, 31]. Hutton et al., showed that the expression of a single protein, CD105, can easily and stably identify pro-tumorigenic CAFs, at least in PDAC [29]. However, as CD105 expression varies between cancer types [29], further studies are needed to elucidate whether CD105-negative CAFs are also a marker of immune response in tumors other than PDAC. Krishnamurty and colleagues identified the leucine-rich-repeat-containing protein 15 (LRRC15) as a promising, highly restricted marker of a subpopulation of CAFs with pro-tumorigenic, immunity-suppressing properties [31].

Despite these obstacles, an increasing number of preclinical studies have focused on CAF targeting as a way to improve anti-cancer strategies, and some clinical trials involving CAF targeting agents are already ongoing (reviewed in [96]).

#### CAF depletion

**Fig. 4:**
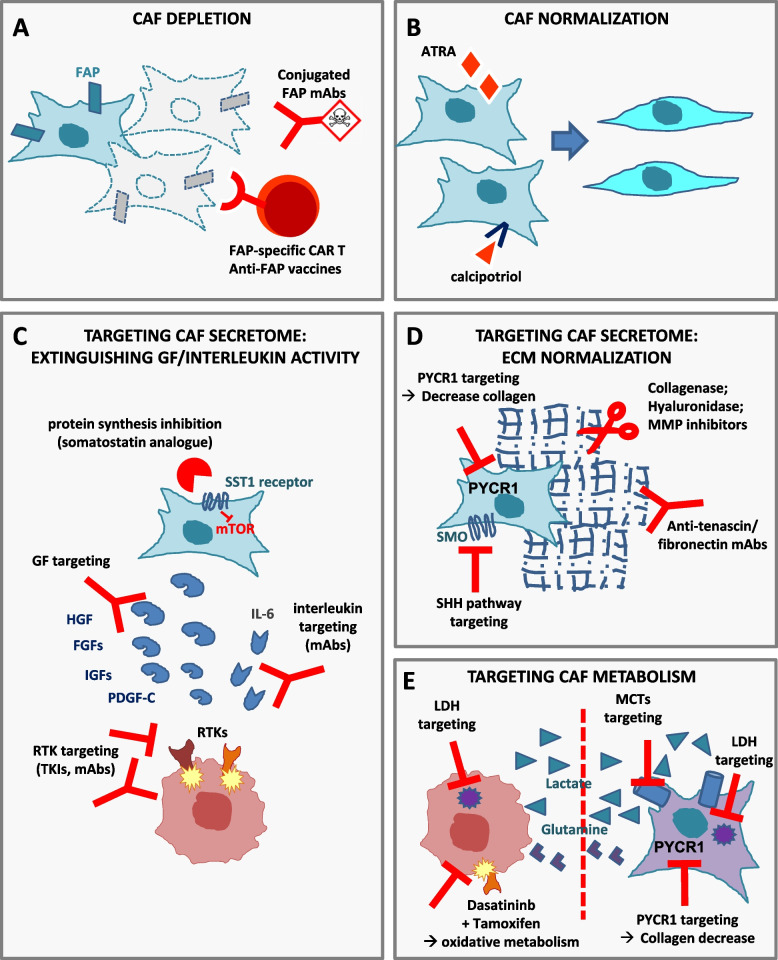
Targeting CAF-mediated resistance. Possible strategies for targeting CAFs comprise: **A** CAF depletion; **B** CAF differentiation towards fibroblasts; **C** targeting growth factors or chemokines released by CAFs; **D** targeting ECM components; **E** interrupting (dashed red line) the metabolic interplay between CAFs and tumor cells. FAP: fibroblast activating protein; ATRA: all-trans-retinoic acid; SST: somatostatin; GF: growth factors; RTKs: receptor tyrosine kinases; TKIs: tyrosine kinase inhibitors; mAbs: monoclonal antibodies; ECM: extracellular matrix; SHH: sonic hedgehog; SMO: smoothened; LDH: lactate dehydrogenase; MCTs: monocarboxylate transporters

Some groups have developed strategies to deplete CAFs (Fig. 4A). The genetic CAF depletion in transgenic mice using fibroblast activating protein (FAP) promoter-driven diphtheria toxin receptor [97] or αSMA-thymidine kinase [27] led to contradictory results as in the first case pancreatic ductal adenocarcinoma growth was slowed down [97] while, in the second case, it became more aggressive and invasive, leading to shorter animal survival [27]. It has to be noted that, based on the results obtained by Öhlund et al., αSMA targeting might preferentially eliminate myCAFs, while leaving other more pro-tumorigenic CAF populations unaffected [20]. However, in both these studies [27, 97], CAF depletion allowed a better immune control of tumor growth and synergized with immunotherapy, opening the possibility for a clinically relevant window of opportunity with anti-CAF compounds. Similarly, McAndrews et al. recently showed that genetic depletion of FAP+ CAFs increased PDAC survival, while depletion of αSMA+ CAFs decreased it [30]. Always using transgenic mice models, Krishnamurty and colleagues selectively depleted the LRRC15+ CAF subpopulation in PDAC, and this was sufficient to significantly slow tumor growth and restore CD8+ T cell functions, increasing response to immunotherapy [31]. Since LRRC15+ CAF formation depends on TGFβ receptor 2 signaling [21], this opens the attractive possibility to use of TGFβ inhibitors to overcome CAF-mediated resistance to cancer immunotherapy.

Different pharmacological CAF-targeting treatments have been developed, such as anti-FAP monoclonal antibodies conjugated with a tubulin-binding maytansinoid [98], anti-FAP antibodies labeled with β-emitting radionuclides [99] or FAP-targeting immunotoxins [100, 101]. Despite promising results in the preclinical setting, where anti-FAP antibodies reduced tumor growth [99] and overcame resistance to chemotherapy in animal models [101], these strategies failed in early phase II studies due to limited ability of the sole anti-FAP antibody of reducing metastatic colorectal cancer burden in patients [102]. DNA vaccines against FAP [103] and FAP-specific CAR-T cells are under development [104, 105] even if, so far, only in the preclinical setting and with contradictory results [106, 107]. In a different perspective, monoclonal antibodies targeting FAP have also been developed as anti-cancer drugs for the delivery of bioactive compounds, such as pro-inflammatory cytokines, not aimed at depleting CAFs but to exploit CAFs as ‘TME specific antigen’ to locally boost the immune response. An example of these antibody-cytokine fusion molecules is represented by the anti-human FAP monoclonal antibody 7NP2 linked to interleukin (IL)-12, which showed encouraging preclinical results [108]. Concerning the recent identification of CD105 as a marker of pro-tumorigenic CAFs in PDAC [29], further research will be required to determine the best way to target the CD105-positive CAFs, thereby specifically depleting the pro-tumorigenic CAF subpopulation while still preserving the tumor-restraining one.

#### CAF normalization

Another strategy to target CAF pro-tumorigenic functions is to revert CAFs from the active to a quiescent state or even to switch their pro-tumorigenic phenotype to a tumor-suppressive one (Fig. 4B). Currently, CAF pharmacological reprogramming has been achieved in specific tumor contexts only, such as in pancreatic ductal adenocarcinoma (PDAC). In PDAC models, treatment with retinoic acid or with the vitamin D receptor ligand calcipotriol induced quiescence of pancreatic stellate cells and profound stromal remodeling, leading to decreased aggressiveness of the surrounding cancer cells and increased response to chemotherapy [109, 110]. CAF normalization would likely provide preferable and safer therapeutic opportunities than CAF depletion, but further preclinical evaluation is required to test its feasibility and clinical translatability.

#### Targeting the CAF secretome

Given the difficulties associated with CAF depletion or reprogramming, at present the most feasible strategy is the targeting of CAF-released factors functionally involved in tumorigenesis and drug resistance (Fig. 4C, D). The broadest approach in this sense is that reported by Duluc and colleagues, who pharmacologically inhibited global protein synthesis in CAFs using a somatostatin analog that, binding the sst1 somatostatin receptor selectively expressed by CAFs, targeted the mTOR-4E-BP1 pathway in these cells, overcoming in this way chemotherapy resistance in PDAC models [111].

Concerning the production of ECM proteins, some attempts have been made to reduce the release of collagen or hyaluronan: the angiotensin receptor blocker losartan, primarily used to treat high blood pressure, was repurposed as a modulator of the tumor extracellular matrix and reduced matrix stiffness in PDAC and breast cancer models, thereby improving drug delivery [112]. Increased chemotherapy efficacy has also been obtained through enzymatic ablation of hyaluronan by recombinant hyaluronanidase [113, 114] or through iodine-131 labeled antibodies targeting tenascin-C [115]. As sonic hedgehog signaling promotes CAF matrix production, sonic hedgehog targeting decreased PDAC desmoplasia and increased tumor response to chemotherapy, anti-angiogenic therapies [116] and cetuximab [117]. As concerns matrix-metalloproteinases targeting, despite several promising results in preclinical models, all the phase III clinical trials performed so far have failed to reach their primary endpoints, even if novel compounds are emerging [118].

Another possibility is to block CAF-produced chemokines, such as CXCL12 [97], or to target growth factors released by CAFs or their receptors on tumor cells. Given the large amount of preclinical data convincingly proving the causative role of HGF in drug resistance, targeting stromal HGF (or its tyrosine-kinase receptor MET expressed on tumor cells) is predicted to counteract tumor resistance. MET inhibition has been evaluated in several clinical trials because *MET* gene amplification is a predictor of response to anti-MET compounds [119]. However, none of these trials were designed to block HGF/MET-driven resistance to other therapies. Despite the encouraging results of a phase II trial [120], a large, randomized phase III trial evaluating onartuzumab (a MET monoclonal antibody affecting HGF-MET binding) in combination with erlotinib in NSCLCs bearing MET overexpression did not confirm the findings of an earlier phase II study [121]. These negative results might be at least partially explained by the fact that patients were not selected for EGFR mutational status, which is required to identify patients sensitive to erlotinib [121].

#### Targeting CAF metabolism

In CAF-mediated breast cancer resistance to tamoxifen, the altered metabolic cross-talk sustaining drug resistance was overcome by targeting CAFs with dasatinib, a multi-tyrosine kinase inhibitor blocking, among the others, PDGFR signaling (from which CAFs are strongly dependent). The combination of tamoxifen plus dasatinib normalized both tumor glucose uptake and mitochondrial activity, reducing ROS formation, and thus interrupting the vicious metabolic cycle in which resistant tumor cells exploit oxidative stress to extract nutrients and high-energy metabolites from adjacent CAFs [90] (Fig. 4E).

As previously mentioned, also lactate mediates adaptive resistance to certain targeted agents, by inducing HGF overproduction in CAFs [37]; accordingly, genetic or pharmacological targeting of molecules involved in the lactate axis, such as lactate dehydrogenase (LDH) or the lactate importer MCT1, overcame resistance in animal models [37]. These preclinical data may have important therapeutic implications, as compounds targeting lactate metabolism have been investigated in several preclinical trials and are currently in clinical development (reviewed in [122]), as well as MCT1 inhibitors (NCT01791595). In the near future, new possible applications for LDH and MCTs inhibitors, in combination with targeted agents, might be investigated to bypass the onset of resistance (Fig. 4E). Finally, as reported above, Kay et al. recently demonstrated that proline synthesis via PYCR1 is a crucial regulator of enhanced collagen production by CAFs. Targeting PYCR1 in CAFs reduced tumour collagen deposition *in vitro* and *in vivo* and was sufficient to reduce tumour growth and metastasis [94]. PYCR1 is a particularly promising metabolic vulnerability, as it is among the most overexpressed genes across tumor types [123]. Even if not directly evaluated by the authors, we can foresee that PYCR1 targeting could be a useful strategy to bypass collagen-mediated resistance (Fig. 4D, E).

## Conclusions

Based on the numerous pro-tumorigenic functions of CAFs, many preclinical and clinical studies have focused on targeting these stromal cells to directly impact on tumor growth and disease progression. However, the vast majority of these studies failed. Which are the possible reasons of this failure? On one side, we still lack specific biomarkers of CAFs to exclusively target them. Another explanation could rely in the high heterogeneity of CAF functions, that sometimes are even anti-tumorigenic. If both pro- and anti-tumorigenic CAFs are present in the same tumor and we indiscriminately target them, the treatment could be inefficient, if not deleterious. Finally, hitting CAFs alone might be insufficient to obtain a significant clinical benefit, as pro-tumorigenic CAFs can favor tumor progression but, likely, they are not strictly required for tumor growth and survival, i.e tumor cells are not ‘addicted’ to CAF presence. On the contrary, a possible window of opportunity might rely on the role played by CAFs in drug resistance. Indeed, the best results obtained so far by CAF targeting were those in combination with other drugs (that, until now, have mostly been chemo- and immune-therapies). In this context, investigating the combined effect of molecular therapies directed against cancer cells and CAF-targeting drugs might help overcome the big issue of primary and acquired drug resistance, eventually improving patient survival. To this aim, *ad hoc* clinical studies should be designed, including endpoints that specifically and objectively evaluate CAF status during therapy.

## Data Availability

Data are available upon reasonable request to the corresponding author.

## Abbreviations

CAF: Cancer-associated fibroblast
ECM: Extracellular matrix
TME: Tumor microenvironment
PDAC: Pancreatic ductal adenocarcinoma
αSMA: Alpha smooth muscle actin
NSCLC: Non-small cell lung cancer
GEMM: Genetically engineered mouse models
MMP: Matrix metalloproteinase
HGF: Hepatocyte growth factor
FGF: Fibroblast growth factor
ESCC: Esophageal squamous cell carcinoma
IHC: Immunohistochemistry
TKI: Tyrosine-kinase inhibitor
lncRNA: Long non-coding RNA
IGF: Insulin-like growth factor
PDGF: Platelet-derived growth factor
NRG-1b: Neuregulin-1b
NNMT: Methyltransferase nicotinamide N-methyltransferase
ADT: Androgen signaling deprivation therapy
FAP: Fibroblast activating protein
ER: Estrogen receptor
LDH: Lactate dehydrogenase
myCAFs: Myofibroblastic CAFs
iCAFs: Inflammatory CAFs
apCAFs: Antigen-presenting CAFs
